# Hepatic Abscess following Yttrium-90 Radioembolization in Patients with Surgical Bilioenteric Anastomosis or Compromised Sphincter of Oddi: A Tertiary Cancer Center Experience

**DOI:** 10.3390/curroncol29100553

**Published:** 2022-09-28

**Authors:** Kevin N. Agahi, Armeen Mahvash, Mohamed E. Abdelsalam

**Affiliations:** 1 Department of Student Affairs, Baylor College of Medicine, Houston, TX 77030, USA; 2 Department of Interventional Radiology, The University of Texas MD Anderson Cancer Center, Houston, TX 77030, USA

**Keywords:** cholangitis, infection, liver abscess, HCC, radioembolization

## Abstract

*Purpose*: We describe our experience with the development of hepatobiliary infection in patients with prior surgical, percutaneous, or endoscopic biliary interventions who are receiving transarterial radioembolization (TARE) with yttrium-90 (^90^Y) for primary or metastatic hepatobiliary lesions. *Methods*: Records of 15 patients with a history of prior biliary intervention and liver malignancy subsequently treated with TARE at the participating medical center from November 2009 to September 2015 were reviewed. The primary endpoint was the development of a hepatic abscess or cholangitis in a patient after radioembolization. *Results*: A total of 15 patients comprising 9 men and 6 women, with a median age of 49 years (range 30–73), underwent 17 TARE with ^90^Y procedures. Of the 15 patients, 2 (13.3%) of them developed a hepatobiliary infection. A single patient (6.6%) developed a hepatobiliary abscess. *Conclusion*: Our study shows a low incidence rate of hepatic abscess following TARE in patients with prior biliary intervention.

## 1. Introduction

Hepatic radioembolization, and more specifically transarterial radioembolization (TARE) with yttrium-90 (^90^Y), has gained wide acceptance as a locoregional therapy for hepatocellular carcinoma (HCC) and liver metastases [1]. A large phase 2 study has demonstrated a low overall complication rate (8.1% of patients had pyloric or duodenal ulcers) with hepatic radioembolization [1]. These complications can include various biliary complications, direct radiation toxicity to the liver, and non-target radiation injuries such as gastrointestinal ulceration, pancreatitis, and radiation pneumonitis. The biliary complications, as reported by Atassi et al. [2], include biliary necrosis, biloma, cholecystitis, biliary stricture, and abscess. Among these, hepatic abscess is one of the more serious because it can lead to sepsis and an increased risk of periprocedural death. Besides the increased risk of death, hepatic abscesses can impair recovery in patients with advanced malignancies or potentially exclude patients from other future courses of treatment [3,4,5,6]. An isolated case report of liver abscess following radioembolization was reported by Mascarenhas et al. [3]. Liver abscess formation is not unique to ^90^Y radioembolization. It has been described in other locoregional therapies, including transcatheter arterial chemoembolization (TACE), bland hepatic artery embolization, percutaneous thermal ablation, and percutaneous intratumoral injection [7,8,9,10]. A known risk factor for the development of a hepatic abscess following any of the aforementioned locoregional treatments is a prior biliary intervention resulting in incompetence or absence of the sphincter of Oddi in cases with a biliary enteric anastomosis [11]. Its incidence is significantly higher in patients with a history of surgical or endoscopic/percutaneous biliary interventions. Several studies have investigated different antibiotic regimens to minimize this risk [4,12,13,14,15,16,17,18,19]. While several chemoembolization studies have reported the incidence of liver abscess in patients with prior biliary interventions, few have reported its incidence in patients undergoing radioembolization. Recently, studies have explored the use of antibiotic prophylaxis to minimize similar risks associated with TARE in patients with a history of biliary intervention(s). A multi-institutional retrospective study by Devulapalli et al. demonstrated that no significant risk was associated with the development of hepatobiliary infection after ^90^Y radioembolization treatment in patients with a history of biliary intervention (HR = 2.00; 95% CI = 0.48, 8.37; *p* = 0.34) [20]. Herein, we describe our experience with radioembolization in patients with primary or metastatic hepatobiliary lesions who had undergone prior surgical, percutaneous, or endoscopic biliary interventions. The primary endpoint of our study was the development of a hepatic abscess or cholangitis in a patient after radioembolization. 

## 2. Materials and Methods

### 2.1. Patient Population

This retrospective study was approved by the MD Anderson Institutional Review Board in compliance with the Health Insurance Portability and Accountability Act. All patients provided written and informed consent for the procedure. A total of 15 patients (17 Radioembolization procedures) treated at our institution, a tertiary cancer center, between November 2009 and September 2015 met our inclusion criteria. The study includes patients at the participating medical center, with a history of bile duct intervention and subsequently receiving TARE treatment. No participants were excluded from the study.

### 2.2. Antibiotic Prophylaxis

All patients received a prescribed prophylactic antibiotic regimen based on the findings of Patel et al. [14]. Patients received 500 mg of oral metronidazole (Flagyl; Teva, Petach Tikva, Israel) twice daily and 500 mg of levofloxacin (Levaquin; Ortho-McNeil, Raritan, NJ, USA) daily beginning 2 days prior to radioembolization and continuing until 2 weeks after the procedure. The patients also received a bowel preparation, which entailed receiving 1 g of erythromycin base (Abbott Laboratories, North Chicago, IL, USA) and 1 g of neomycin (Teva) orally at 1:00 pm, 2:00 pm, and 11:00 pm the day before the procedure. All the patients tolerated the regimen well except one patient who developed diarrhea. This patient was switched to daily 400 mg of moxifloxacin (Avelox; Bayer HealthCare, Berlin, Germany). 

### 2.3. Yttrium-90 Radioembolization

Every patient was screened prior to the procedure by the same interventional radiologist (A.M.), who performed all aspects of each procedure. Mapping diagnostic angiograms were performed to evaluate the vascular anatomy, embolize any extrahepatic arteries, and determine the hepatopulmonary shunt fraction on single-photon emission computed tomography/computed tomography (CT) scans after technitium-99m macroaggregated albumin injection. 

Of the 17 radioembolizations performed, 13 were performed in the more conventional manner by embolizing any extrahepatic arteries with microcoils. The remaining four were performed using the balloon occlusion technique as previously described [21,22]. The two patients with HCC underwent radioembolization with TheraSphere ^90^Y glass microspheres (MDS Nordion, Ottawa, ON, Canada), while 13 patients underwent radioembolization with SIR-Spheres ^90^Y resin microspheres (Sirtex Medical Limited, Lane Cove, Australia). The ^90^Y glass microsphere dose was calculated based on the volume of the liver to be treated, while the ^90^Y resin microsphere dose was calculated using the body surface area and estimated tumor burden [1].

In addition to radioembolization, two patients (one of whom underwent two radioembolization procedures) also received bland embolization for additional sites of disease that could not be safely treated with radioembolization. The first of these patients received embolization of a right phrenic artery supplying an HCC using 40-µm and 100-µm Embozene microparticles (CeloNova BioSciences, San Antonio, TX, USA) followed by Gelfoam (Pharmacia and Upjohn Company, Kalamazoo, MI, USA) until complete stasis was achieved within the vessel. The second patient received embolization of an extrahepatic internal mammary vessel using 100-µm to 300-µm Contour PVA particles (Boston Scientific, Natick, MA, USA) followed by 300- to 500-µm and 500- to 700-µm PVA particles. A Gelfoam pledget was also used to complete the embolization to stasis.

### 2.4. Follow-Up

No patients were lost to follow-up. Patients were followed-up with for a median of 284 days (range 29–2258 days, mean: 445.4 days). Each patient had either a routine clinical follow-up or phone call 30 days after the intervention. Patients then had routine cross-sectional imaging scans after 2 months that were then repeated every 3 months. 

## 3. Results

A total of 15 patients were included in this study. A total of 17 radioembolization procedures were performed on the 15 patients, 9 men and 6 women, with a median age of 49 years (range 30–73). Two patients underwent a second radioembolization. The median prescribed activity was 52.2 mCi (1.931 GBq), and the median delivered activity was 50.6 mCi (1.872 GBq). Some of the patients received the TARE concurrent with other systemic therapies (*n* = 8) while some of the patients received the TARE following other systemic therapies (*n* = 7, Median 2 months, (range 1–4 months, mean = 2.34 months)

All patients had previously undergone some form of biliary intervention that resulted in a compromised sphincter of Oddi. Namely, these interventions were seven biliary-enteric anastomoses (four Whipple procedures, two hepaticojejunosomies, and one pancreaticojejunostomy), six biliary stents (five endoscopic internal and one internal/external biliary stent), one sphincterotomy, and one external drainage catheter in the tumor, which was placed for a non-sterile biliary tree and resulted in a compromised sphincter of Oddi. The patient who underwent a pancreaticojejunostomy still had an intact sphincter of Oddi; however, the pancreatic-enteric anastomosis due to the distal pancreatectomy allowed normal bowel flora to seed the biliary system in a similar fashion as the other patients. Patient demographics, biliary interventions, and the nature of each malignancy are summarized in Table 1.

Four grade 3 adverse events, assessed using the Common Terminology Criteria for Adverse Events (CTCAE) version 4.0, occurred within the first 30 days. These included nausea and vomiting in two patients that required hospital admission with intravenous fluid replacement, abdominal pain in one patient, and fever in one patient. The patient’s fever was worked up with no infectious source identified and ultimately deemed to be related to tumor lysis. 

**Figure 1:**
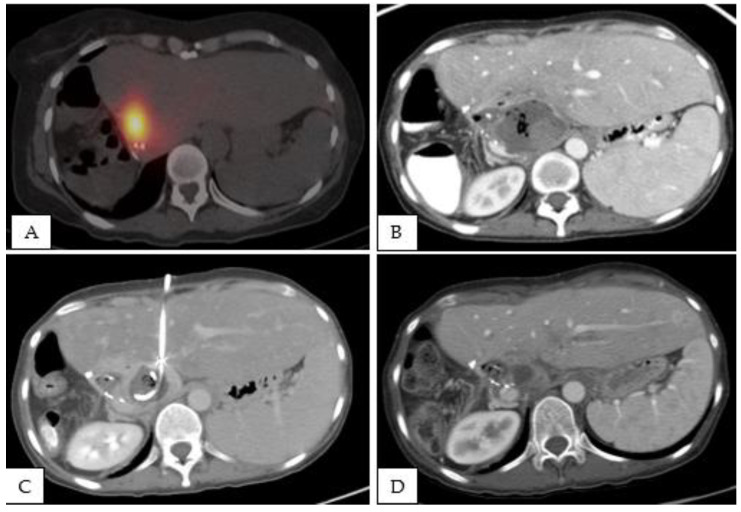
A 48-year-old woman with hepatocellular carcinoma and a history of hepaticojejunostomy received TARE. (**A**) SPECT CT following the ^90^Y treatment. The patient presented with worsening abdominal pain, fever, and mild leukocytosis 71 days after ^90^Y glass radioembolization. A contrast-enhanced CT scan of the abdomen and pelvis showed a 7.2 × 4.5 cm abscess (**B**). She was treated with intravenous antibiotics and a CT-guided percutaneous drain placement (**C**). The abscess drain was removed 31 days after discharge. After this, evaluation under fluoroscopy demonstrated that the small residual abscess cavity (**D**) was freely communicating with the biliary system and emptying through the hepaticojejunostomy.

Between 30 and 90 days after the intervention, there were three CTCAE grade 3 events. These included one hepatic abscess, one episode of cholangitis, and one episode of sepsis with no identified source. The single hepatic abscess was diagnosed 71 days after ^90^Y glass radioembolization. This patient had a fibrolamellar HCC that received right hepatectomy with hepaticojejunostomy in an outside institution. One year later, the patient developed a new lesion in the left lobe of the liver. She received systemic therapy and then decided to seek a second opinion in our institution. After discussion in a multidisplinary tumor board, the patient received TARE. Table 2 summarizes the lab values before and 2 weeks after the TARE. Then, 71 days following TARE, the patient presented with worsening abdominal pain, fever, and mildly elevated white blood cell count at 11.8 K/µL. A contrast-enhanced CT scan of the abdomen and pelvis identified a fluid collection in the liver, consistent with an abscess. She was admitted to the hospital for intravenous antibiotics and had a CT-guided percutaneous drain placed. She was discharged after 14 days, and the abscess drain was removed 31 days after discharge. After this, evaluation under fluoroscopy demonstrated that the small residual abscess cavity was freely communicating with the biliary system and emptying through the hepaticojejunostomy (Figure 1). The patient had no lasting or recurrent symptoms related to the treated hepatic abscess. 

The episode of cholangitis occurred in a patient with an existing endoscopically placed internal biliary stent due to a hilar cholangiocarcinoma. The cholangitis resolved after replacement of the biliary stent by endoscopic retrograde cholangiopancreatography, leading us to suspect the cholangitis was related to obstructed biliary drainage rather than TARE. Finally, the episode of sepsis was treated at an outside local hospital with intravenous antibiotics, necessitating hospitalization for approximately 1 week. Follow-up staging imaging at our institution revealed no hepatic abscess or other sources of the infection. 

At the time of data analysis (February 2020), 9 of the 15 patients who underwent 17 total interventions are still alive, while the 6 other patients died an average of 328 days after treatment. The two patients treated for HCC are still alive, with one of them ultimately receiving a liver transplant after adequate disease control with TARE. 

## 4. Discussion

In this study, 2 of 17 (11.8%) TARE procedures resulted in the development of a hepatobiliary infection following and directly related to the procedure. Of the 15 patients, 2 patients (13.3%) developed a hepatobiliary infection. A single patient (6.6%) developed a hepatobiliary abscess. This is greater than the reported rate (0–2%) in patients undergoing TARE with an intact ampulla of Vater [2,19,20]. Post-embolization liver abscesses are associated with very high morbidity and mortality [4,5,6]. 

In a retrospective study reported by Kim et al., six of the seven patients (85.7%) who had previously undergone a Whipple procedure developed an abscess following TACE [12]. Among the patients without biliary enteric anastomosis, only 1 of 150 patients (0.7%) (or 1 of 383 procedures (0.3%)) developed an abscess [12]. Patel et al. studied the effect of aggressive antibiotic prophylaxis during chemoembolization on the incidence of liver abscess. In their study, the incidence rate of liver abscess per procedure with a standard antibiotic regimen was 42.8% in patients who had previously undergone biliary interventions. However, its incidence rate was reduced to 12.5% with an aggressive antibiotic regimen in patients with similar characteristics [14]. We used the antibiotic regimen published by Patel et al., aiming to minimize the risk of liver abscess. All our patients received aggressive antibiotic prophylaxis (48 hours before and up to 2 weeks after therapy) as well as bowel preparation with erythromycin and neomycin. With these precautions in place, only 1 of our 17 radioembolizations was complicated by a hepatic abscess. Therefore, the incidence rate of liver abscess in our study is comparable to the one found in the study done by Patel et al. in patients receiving chemoembolization [14]. 

Mezhir et al. performed 2167 small-particle bland embolizations of the liver in 1255 patients [19]. Fourteen patients (1.1%) developed a hepatic abscess. Of these 14 patients, only 1 patient had not previously undergone a biliary procedure.

It is thought that patients with prior biliary interventions are prone to developing enteric bacterial colonization of the biliary tree after TARE procedures. Arterial supply to the biliary tree occurs via the peribiliary capillary plexus that arises from the hepatic artery, which measures approximately 30 µm in diameter. Therefore, any form of embolization has the potential to cause biliary ischemia. The combination of bacterial colonization and biliary ischemia appears to be a plausible cause for liver abscess formation [11].

The embolic effect of ^90^Y microspheres is distinct from that of conventional TACE utilizing lipiodol and Gelfoam pledgets [7,9,12,13,14,15,16,17]. The peribiliary capillary plexus would appear to have a greater risk of being embolized by the TACE using Lipiodol given that the primary end point is complete embolization to induce ischemia, not the case in TARE

Given their difference in a specific activity, fewer glass microspheres are necessary than embolic resin microspheres to deliver a similar dose of radiation. Assuming the embolic effect with resultant ischemia to the peribiliary capillary plexus was the only variable leading to abscess formation, one might hypothesize that the resin microspheres are associated with a greater risk of abscess formation given the higher number of microspheres used to achieve the same dose. However, the direct radiation effect on the peribiliary capillary plexus likely plays an equally important role in the possibility of developing an abscess. De Jong et al. reported the development of a hepatic abscess following whole liver irradiation in a patient who had undergone a prior Whipple procedure [8]. 

Of the 14 other patients treated, 1 other patient was treated with the glass microspheres while the remaining 13 were treated with the resin microspheres. Given the small patient cohort in this study, comparing both devices is not feasible.

Our study has some limitations. First, several limitations are associated with its retrospective nature (i.e., causation cannot be determined, only association and confounding variables that were present for each case may not have been recorded). The second limitation is the heterogeneity of the patient population with respect to tumor nature, tumor biology, prior therapies, and their timing in relation to the TARE. Third, our study had a small sample size, which precluded statistical analysis. A larger multicenter study would accurately estimate the safety of TARE in patients with a prior biliary intervention. However, these early results show that TARE may have a similar safety profile to the other, more well-studied locoregional therapies. 

## 5. Conclusions

In conclusion, our study shows a low incidence rate of hepatic abscess following TARE in patients with prior biliary intervention. Using an aggressive antibiotic regimen may have decreased the number of infectious complications. Larger future studies are needed to elucidate the safety of TARE in this high-risk patient population. 

## Figures and Tables

**Table 1:** Patients’ characteristics.

Demographics	
**Median age (y)**	49 (range 30–73)
Sex	
M	9 (60)
F	6 (40)
**Nature of Malignancy**	
Primary liver tumor	6 (40)
Hepatocellular carcinoma	4 (27)
Cholangiocarcinoma	2 (13)
Metastatic disease	9 (60)
Adenocarcinoma	1 (6.7)
Neuroendocrine tumors	7 (47)
Ameloblastoma	1 (6.7)
**Biliary intervention**	
Biliary-enteric anastomosis	7 (47)
Endoscopic stent	6 (40)
Biliary drainage catheter	1 (6.7)
Sphincterotomy	1 (6.7)
**Concurrent systemic chemotherapy**	
Yes	8 (53)
No	7 (47)
**Prior liver resection or ablation**	
Yes	2 (13)
No	13 (87)
**Prior chemoembolization or** **bland embolization**	
Yes	2 (13)
No	13 (87)
**ECOG performance status**	
**0**	12 (80)
**1**	3 (20)
**2**	0 (0.0)
**3**	0 (0.0)

**Table 2:** Laboratory values before and after the TARE for the patient who developed Liver abscess (Child Pugh A).

Lab Value	Before TARE	After TARE (2 Weeks)
Aspartate Transaminase (U/L)	34	45
alanine transaminase (U/L)	19	25
Alkaline phosphatase (U/L)	148	207
Bilirubin (md/dL)	0.5	0.8
Albumin(g/dL)	4.4	4.5
Prothrombin Time (sec)	14.2	14.9
Platelets (K/μL)	252	208
Hemoglobin (gm/dL)	14.8	13.5
White blood cells (K/μL)	5.5	4.3

## Data Availability

The data presented in this study are available on request from the corresponding author. The data are not publicly available due to institutional restrictions.
